# Documenting two decades of longitudinal changes to a community-engaged obesity prevention intervention for Native American families to facilitate scale-up

**DOI:** 10.1093/tbm/ibag056

**Published:** 2026-08-28

**Authors:** Emily J Tomayko, Paul A Estabrooks, Teresa M Warne, James L Merle, Alexandra K Adams

**Affiliations:** Department of Food Systems, Nutrition, and Kinesiology, Montana State University, Bozeman, MT, United States; Center for American Indian and Rural Health Excellence, Montana State University, Bozeman, MT, United States; Department of Health and Kinesiology, College of Health, University of Utah, Salt Lake City, UT, United States; Center for American Indian and Rural Health Excellence, Montana State University, Bozeman, MT, United States; Department of Population Health Sciences, School of Medicine, University of Utah, Salt Lake City, UT, United States; Center for American Indian and Rural Health Excellence, Montana State University, Bozeman, MT, United States

**Keywords:** implementation science, health promotion, program evaluation, American Indian/Alaska Native, health behavior, pediatric obesity

## Abstract

**Background:**

Community-based participatory research (CBPR) interventions frequently undergo adaptation as they move from efficacy studies to dissemination; yet long-term adaptations are rarely documented in ways that support replication or scale-up.

**Purpose:**

To demonstrate a structured approach for retrospectively documenting longitudinal adaptations to *Healthy Children, Strong Families* (HCSF)—a culturally grounded, evidence-based obesity prevention intervention for Native American families—as it evolved into *Turtle Island Tales*.

**Methods:**

We conducted a retrospective review of permanent records generated between 2006 and 2026 spanning three major program iterations: *HCSF1*, *HCSF2*, and *Turtle Island Tales*. Guided by the Framework for Reporting Adaptations and Modifications to Evidence-based Interventions (FRAME), the implementation-strategy extension FRAME-IS, and the Adaptome, adaptations were categorized as changes to intervention governance, content, structure, or strategy. Data sources included intervention materials, study protocols, quantitative and qualitative trial data, meeting minutes, and emails.

**Results:**

Adaptations were layered and interdependent and were documented as preserving core functions while supporting intended implementation outcomes (e.g. acceptability, feasibility, reach, adoption, sustainability). Governance adaptations reflected shifts from more centralized, CBPR-grounded adaptation and implementation processes to greater local decision-making by community implementers. Content adaptations included greater integration of traditional knowledge and new lessons addressing sleep, emotional regulation, and family traditions. Structural adaptations involved delivery changes aligned with community systems and development of culturally resonant branding and characters. Strategy adaptations included opt-out classroom-based recruitment, embedding implementation within existing community roles, and revised training materials.

**Conclusions:**

Organizing adaptations into governance, content, structure, and strategy provides a pragmatic approach for documenting flexibility across long-term CBPR partnerships and may offer considerations for documentation of intervention adaptations in similar settings.

**Clinical Trial information:**

The Clinical Trials Registrations #NCT01776255, NCT06298149.

## Teaser

Over nearly two decades, a family-based wellness program developed with Native American communities evolved to better fit schools and community settings while preserving its core functions. This study shows how effective programs can change over time without compromising the elements needed to achieve their intended outcomes.

Implications **Practice:** For community implementers, this work illustrates how evidence-based programs can be adapted by changing how and where they are delivered to fit existing routines and systems, while keeping the program’s core lessons and intended outcomes intact.

**Policy:** Funders and policymakers should support flexible, longer-term funding that allows academic and community partners to adapt delivery approaches over time while maintaining accountability to core intervention functions.

**Research:** Prospectively tracking layered adaptations and their rationale over time will facilitate better understanding of how changes in delivery structure shape implementation strategies, reach, and sustainability in long-term CBPR interventions.

## Introduction

Native American communities have long relied on many strengths to counter historic trauma, racism, rural isolation or urban loss of community connections, and persistent poverty [1] (Note: Indigenous peoples may prefer different terminology that will vary globally, within the geographic constraints of a country, by region, by tribe, or individually within a tribe or community. We herein use the term ‘Native American’ to describe the first peoples of the geographic boundary of the United States.). Still, these challenges have resulted in significant health differences compared to non-Hispanic White populations [2–4] and have contributed to Native American people having the lowest life expectancy of any racial/ethnic group in the United States [5]. Obesity is of particular concern among Native American communities and affects individuals across the lifespan [6]. These rates are consequential among young children, as obesity often persists through adolescence into adulthood [7] and increases the risk of cancers and chronic diseases [8].

Within this context, CBPR approaches have been used to cocreate health interventions that align with Native American community priorities and resources. CBPR focuses on intervention designs that fit community contexts while considering where and by whom an intervention is adopted, implemented, and maintained. Because of this dual focus, CBPR studies also may contribute to advancing community-engaged dissemination and implementation science (CEDI), which examines how evidence-based interventions are adopted, implemented, and maintained in real-world settings [9].

*HCSF* is an obesity prevention intervention for families with young children that was cocreated and tested through a CBPR approach with multiple Native American communities [10–14]. *HCSF* was designed to promote positive family health behaviors across diet, physical activity, screen time, sleep, and emotional regulation. The intervention was organized around 12 monthly kits that include parent lessons, children’s books, recipes, activities, and support items.

**Figure 1:**
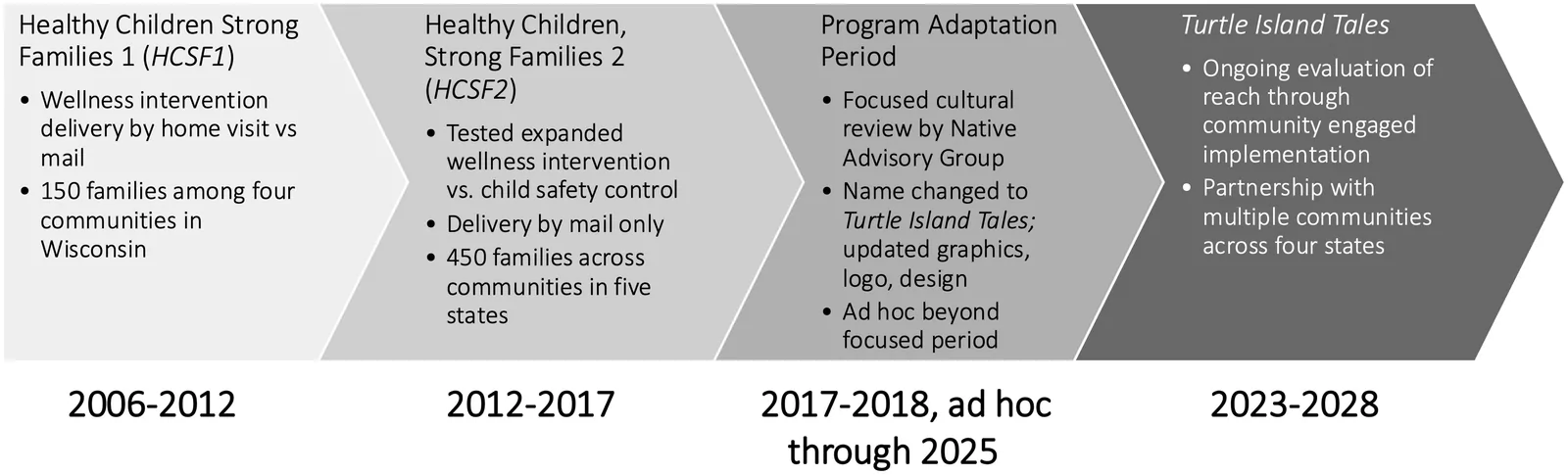
General overview and timeline of three major program iterations and focused adaptation period.

After collaboratively developing intervention materials, the initial clinical trial (*HCSF1*, 2006–12, National Institutes of Health/National Heart, Lung, and Blood Institute), compared intervention delivery via home visits versus delivery by mail in partnership with four Wisconsin tribes (see Fig. 1 for a general timeline) [10–12]. After determining similar effectiveness across home visit and mailed delivery modalities, *HCSF2* (2012–17, National Institutes of Health/National Heart, Lung, and Blood Institute) evaluated the effectiveness of the mailed-only intervention relative to a mailed child-safety control condition across five communities in multiple states [13, 14]. Both *HCSF* trials demonstrated high retention, improved health behaviors, and strong acceptability among families and communities [12, 14]. Partner communities also emphasized a strong commitment to the well-being of future generations. A subsequent adaptation period (2017–18, *ad hoc* through 2025) focused on highlighting community strengths and preparing the intervention (now referred to as a “program”) for broader dissemination. During this phase, the program was renamed *Turtle Island Tales*. An ongoing dissemination and implementation study (2023–28, National Institutes of Health/National Cancer Institute) is evaluating program reach and adoption across communities [15]; outcome data from this study are not reported here. Rather, this article documents adaptations that occurred across program iterations and during community implementation.

CEDI-aligned approaches that distinguish between core *functions* (e.g. components necessary to achieve intervention outcomes) and *forms* (e.g. activities or channels used to enact those functions) may be useful in reporting adaptations to CBPR interventions [16]. Existing taxonomies for reporting adaptations, such as the expanded Framework for Reporting Adaptations and Modifications to Evidence-based Interventions (FRAME), have been proposed to document specific adaptations, when and how they occurred, whether adaptations were planned or reactive, who proposed and made adaptation decisions, and the category of adaptation (e.g. content, structure) [17]. The Framework for Reporting Adaptations and Modifications to Evidence-based Implementation Strategies (FRAME-IS) extends adaptation reporting to dissemination and implementation strategies by documenting what was modified, the relationship to core functions, rationale, timing, and spread of the modification [18]. In long-term CBPR partnerships, however, the structures through which adaptation decisions are generated, reviewed, approved, and localized also may evolve over time [19]. Tracking these governance adaptations may be especially important in CBPR because shifts in decision authority and local discretion can influence which adaptations occur, whether core functions are preserved, and whether interventions can be sustained in community settings.

CBPR approaches also lend themselves to tracking adaptations through retrospective reviews of permanent records that are gathered during partnership meetings, email communications, and shared iterative versions of interventions and strategies. While these records are rich sources of information, it is also likely that retrospective reviews of intervention records may lack sufficient detail to fully populate existing adaptation framework components, particularly regarding timing, decision-makers, and intent, limiting reliable application across long-term CBPR collaborations [17, 20]. Accordingly, retrospective documentation should be clear about which elements can be reconstructed and which cannot. In this article, we use *HCSF/Turtle Island Tales* as a longitudinal case example to examine how adaptations can be categorized, justified, and linked to core functions during community-engaged scale-up. We also consider how these changes were expected to influence implementation outcomes.

## Methods

### Study design

We conducted a retrospective document review using a directed content analysis approach [21] to identify, categorize, and describe adaptations across three main program iterations: *HCSF1*, *HCSF2*, and *Turtle Island Tales*. Our process was informed by elements from multiple frameworks, including FRAME, to classify adaptations by type, timing, and rationale [17]; FRAME-IS to specify adaptations to implementation strategies [18]; and the Adaptome to consider the cumulative layering of adaptations across program versions [22].

### Data sources and review scope

Adaptations were identified through a structured review of intervention materials, study and Institutional Review Board (IRB) protocols, prior trial quantitative (e.g. dietary recalls, behavioral surveys) and qualitative (e.g. focus groups, participant surveys addressing barriers and facilitators, community input) outcome data, study-related meeting minutes and emails, and records of cultural review across program iterations. The review was limited to documented adaptations that could be identified in these records or confirmed through research team consensus based on the records. Sources were reviewed to identify documented adaptation in intervention governance, content, structure, and strategies.

### Adaptation coding framework

We defined a reportable adaptation as a change to program governance, content, structure, or strategy that was retained across program versions or intentionally implemented across one or more community sites. Minor copyediting, formatting changes, and one-time logistical adjustments that did not alter governance, content, structure, or strategy were not coded as adaptations. The coding matrix was developed deductively from FRAME, FRAME-IS, the Adaptome, and the core function/form distinction. Operational definitions and coding rules for these four categories are summarized in Table 1, and core functions are defined in Table 2.

**Table 1:** Operational definitions and coding rules for adaptation categories.

Adaptation category	Operational definition	Coding rules	Examples from *HCSF1/HCSF2* and *Turtle Island Tales*	Framework
Governance	How adaptation decisions were generated, reviewed, documented, approved, and localized through CBPR partnerships	Coded as governance when the adaptation primarily concerned who participated in the decision-making, how community input was elicited/reviewed, how authority was shared, or how local implementers were given discretion over adaptation decisions	CABs; Native Advisory Group; local implementer decision-making	CBPR/CEDI governance; FRAME/FRAME-IS decision process elements
Content	What families are intended to learn, internalize, or practice; includes health topics, lesson objectives, teachings, narratives, and cultural framing	Coded as content when the underlying educational objective, lesson topic, message, teaching, or cultural framing changed	New lesson topics (“Sleep Tight,” “Feeling Your Feelings,” “Family Traditions”); lesson name changes (“Maintaining Harmony,” “Active Anywhere”); lesson consolidation (“Naturally Sweet”); medicine wheel framing; grandmother quotes	FRAME content modification/cultural tailoring; function/form distinction; Adaptome cumulative adaptation
Structure	How content is packaged, sequenced, branded, delivered, timed, or supported through materials, personnel, modality, and delivery systems	Coded as structure when the form, format, dose, timing, materials, delivery mode, or packaging changes without changing the core educational objective or function	Home visit to mailed delivery; classroom/backpack distribution; school-year timing; branding/logo/color scheme; Igmu and Grandma characters; films; newsletters/local materials	FRAME delivery/context modifications; Adaptome attention to how components are delivered; function/form guidance
Strategy	Actions taken by people, organizations, or systems to improve reach, adoption, implementation quality, engagement, evaluation completion, sustainability, or maintenance	Coded as strategy when the change is an intentional action, workflow, recruitment approach, incentive, training activity, communication channel, or implementation support	Classroom recruitment with opt-out; local partner recruitment roles; training; instructor manual; incentives/raffles; removal/localization of text-message retention support; Facebook-to-Instagram transition	FRAME-IS; ERIC-style definition of implementation strategies as actions; RE-AIM implementation outcomes

Abbreviations: CBPR, community-based participatory research; CEDI, community-engaged dissemination and implementation; FRAME, Framework for Reporting Adaptations and Modifications to Evidence-based Interventions; FRAME-IS, Framework for Reporting Adaptations and Modifications to Evidence-based Implementation Strategies; ERIC, Expert Recommendations for Implementing Change; RE-AIM, Reach, Effectiveness, Adoption, Implementation, and Maintenance Framework.

**Table 2:** Core functions intended to be preserved during adaptations across all program iterations.

Core function	Examples of the core function
Promote family-level wellness behaviors	Lessons and support items addressing fruit/vegetable intake, added sugar, physical activity, screen/sedentary time, sleep, stress/emotional regulation, and family/community traditions as part of holistic family wellness
Support parent/caregiver–child co-engagement	Home-based kits, children’s books, recipes, support items, activity prompts, and school/classroom distribution intended to reach families
Ground the program in Native culture, strengths, and intergenerational knowledge sharing	Inclusion of traditional knowledge, Native authors/illustrators for books and lesson materials where possible, medicine wheel, Grandmother quotes, Grandma/Igmu characters and story world, Seven Grandfather teachings, and family/community traditions lesson
Maintain low-burden access for families	Home visits, mailed kits, school/classroom/backpack distribution, parent-night distribution, summer packets, and limited hybrid home delivery where locally feasible
Support community fit and local ownership	Input from CABs and local implementers; iterative Native Advisory Group review; local implementer discretion for newsletters, delivery events, and evaluation incentives

Governance was defined as a change in how adaptation decisions were made based on who participated, how community input was elicited or reviewed, how decision-making authority was shared, how adaptations were approved, or how local implementers were given discretion to adapt optional components. Content adaptations were defined as changes to lesson topics, teachings, or cultural framing. Moreover, FRAME specifies characterizing the nature of content modifications and their relationship to fidelity and highlights cultural tailoring as a reportable rationale [17]. Structure adaptations were defined as changes to how the intervention content was packaged, sequenced, branded, delivered, timed, or supported through materials, personnel, modality of delivery, and delivery architecture. These align with context- and delivery-level modifications in FRAME and with the Adaptome’s focus on cumulative changes across intervention versions [17, 22]. Finally, consistent with FRAME-IS [18], strategy adaptations were defined as actions or workflows intended to improve dissemination and implementation outcomes, including reach, adoption, implementation, or maintenance.

When adaptations plausibly fit more than one category, the team assigned a primary code based on the closest fit with operational definitions (Table 1). Secondary codes were assigned when an adaptation also supported another category. For example, when a governance adaptation produced changes to lesson topics, delivery modes, or recruitment or implementation supports, those changes were coded secondarily as content, structure, and strategy, respectively.

### Coding, review, and consensus process

Initial coding was conducted jointly, rather than independently, through iterative review by two members of the research team who had been involved with the program across all iterations (E.J.T., A.K.A.). This consensus-based approach was selected because many adaptations were identified through historical program knowledge paired with permanent record review. Secondary review was provided by members of the research team with expertise in dissemination and implementation science (P.A.E., J.L.M.). Differences in inclusion, interpretation, or categorization were discussed by all authors, and final inclusion and categorization were determined by group consensus.

### Data extraction and synthesis

For each adaptation, we extracted the following components, when available: program iteration or timing, planned or reactive status, modification description, rationale/intent, decision source or process, available evidence source, core function preserved, and intended implementation outcome supported. Prior trial outcomes and qualitative findings were used to identify documented rationales for adaptations. We did not attempt to estimate the causal effect of individual adaptations on effectiveness or implementation outcomes. Because this was a retrospective review of program records, we report unavailable elements in the tables when documentation was insufficient. Missing details were not inferred.

## Results

Across HCSF1, *HCSF2*, and *Turtle Island Tales*, we identified community-engaged adaptations in governance, content, structure, and strategy (Table 3).

**Table 3:** Major adaptations across *HCSF*/*Turtle Island Tales* by adaptation category.

Major adaptation	Timing/status^a^	Modification description	Evidence source^b^	Rationale	Decision source	Secondary adaptation category	Core function preserved^c^	Intended implementation outcome supported^d^
**Primary adaptation category: governance**								
CABs	During program development; during *HCSF1* and *HCSF2*/*planned trial governance structure*	CABs supported study design and conduct, community fit, recruitment, and study progression	Study protocols; CAB meeting minutes	Maintain CBPR alignment, community oversight, and local fit during trials	CABs and research team	Strategy	Support community fit and local ownership	Acceptability, feasibility
Native Advisory Group formalized cultural and content review	*Turtle Island Tales*/*planned adaptation governance structure*	A formal five-member group reviewed language, graphics, lesson topics, cultural appropriateness, medicine wheel integration, lesson renaming, and new lesson development	Meeting minutes; material revision history	Strengthen cultural relevance and prepare program materials for broader dissemination	Native Advisory Group recommendations reviewed and incorporated by research team	Content, Structure	Ground the program in Native culture, strengths, intergenerational knowledge sharing	Cultural appropriateness, acceptability, scale-up through reach and adoption
Local implementer discretion expanded for optional/adaptable components	*Turtle Island Tales*/*planned prospective implementation design*	Communities determined whether and how to use newsletters, events to distribute first kits, hybrid delivery variations, and evaluation incentives	Meeting minutes, emails	Increase local fit, ownership and sustainability	Local community implementers/partners with research team support	Structure, Strategy	Support community fit and local ownership	Feasibility, adoption, maintenance
**Primary adaptation category: content**								
Sleep Tight lesson added	Between *HCSF1* and *HCSF2*/*planned*	A child focused sleep hygiene lesson was developed	Quantitative trial data (*HCSF1* dietary recalls)	Address late-night eating patterns observed through dietary recalls and promote sleep as part of family wellness routines	Research team informed by trial findings	None identified	Promote family-level wellness behaviors	Acceptability, relevance (to family routines)
Maintaining Harmony lesson added	Between *HCSF1* and *HCSF2*/*planned*	An adult focused stress management lesson was developed	Qualitative trial data (focus groups)	Address stress as a barrier to family wellness and health behavior change	Research team informed by trial findings	None identified	Promote family-level wellness behaviors	Acceptability, appropriateness,
‘Feelings Your Feelings’ and ‘Family Traditions’ lessons added	Between *HCSF2* and *Turtle Island Tales*/*planned*	A child-focused emotional regulation lesson and a lesson that address family and community traditional practices were developed	Native Advisory Group and community implementer input via meeting minutes, emails	Address child emotional regulation; shift program materials to more asset/strengths based (traditions, traditional practices)	Native Advisory Group, research team	None Identified	Promote family-level wellness behaviors; ground the program in Native culture, strengths, and intergenerational knowledge sharing	Cultural appropriateness, acceptability
Nutrition content was consolidated and reframed	Between *HCSF2* and *Turtle Island Tales*/*planned*	The lessons focused on fruit and added sugar were consolidated into Naturally Sweet; the fast-food focused lesson was removed	Native Advisory Group and community implementer input via meeting minutes, emails	Preserve nutrition content while making room for new lessons, emphasizing nutrient-dense foods rather than restriction	Native Advisory Group, research team	Structure	Promote family-level wellness behaviors	Feasibility, program dose consistency
Cultural framing strengthened across program	Between *HCSF2* and *Turtle Island Tales*/*planned*	Added medicine wheel framing and grandmother quotes/wisdom to each lesson, incorporated grandfather teachings in lesson materials, revised lesson names	Native Advisory Group and community implementer meeting minutes, emails	Integrate traditional knowledge, promote holistic wellness, create clearer cultural framing throughout program	Native Advisory Group, research team	Structure, Governance	Ground the program in Native culture, strengths, intergenerational knowledge sharing	Cultural appropriateness, acceptability
**Primary adaptation category: structure**								
Delivery shifted from home visits to mailed kits	Between *HCSF1* and *HCSF2*/*planned*	HCSF1 tested home visits by community mentors versus mailed kits; HCSF2 adopted mailed delivery as the standard modality	Trial data; implementation experience related to cost and scheduling	Reduce cost and scheduling burden while maintaining program exposure	Research team informed by trial findings, input from CABs/community implementers	Strategy	Maintain low-burden access for families	Feasibility, cost, maintenance
Program identity and materials were redesigned	Between *HCSF2* and *Turtle Island Tales*/*planned*	Program renamed Turtle Island Tales; logo and color scheme developed and applied across materials; Grandma and Igmu characters developed and incorporated into program; short films developed to support program	Participant focus groups, informal conversation notes; Native Advisory group meeting minutes and emails; material revision history	Better reflect diversity of communities across Turtle Island in preparation for broader dissemination; unified branding and colors to increase program recognition and engagement	Native Advisory group, research team, graphic design and film partners	Content, Strategy	Ground the program in Native culture, strengths, intergenerational knowledge sharing; support parent/caregiver-child co-engagement	Cultural appropriateness, scale-up readiness
Distribution shifted to school and classroom settings	*Turtle Island Tales*/*planned*	Program distributed through Head Start/school classrooms via backpacks, cubbies, parent events, summer packets, or limited hybrid home delivery where locally feasible	Local implementer meeting minutes from each community implementation team	Align with community systems (Head Start, school), reduce burden of maintaining individual mailing, support local fit	Community partners with research team support	Strategy, Governance	Maintain low-burden access for families; support parent/caregiver-child co-engagement	Reach, feasibility, adoption, maintenance
Program timing aligned with school year	*Turtle Island Tales*/*planned*	Recruitment and program launch shifted from rolling enrollment to August/September start aligned with school calendar	Local implementation team meeting minutes and emails	Streamline implementation and support community planning	Research team, community partners	Strategy	Maintain low-burden access for families	Feasibility, adoption
**Primary category: strategy**								
Recruitment shifted from community-wide opt-in to classroom-based opt-out	*Turtle Island Tales*/*planned*	Community-wide recruitment through events, flyers, and social media was replaced by classroom-based distribution with opt-out enrollment.	Local implementer meeting minutes, notes, emails	Reduce individual-level recruitment burden, align with school systems	Community partners with research team support	Structure	Maintain low-burden access for families	Reach, adoption, feasibility
Implementation roles embedded in existing community systems	Across versions, expanded in *Turtle Island Tales*/*planned*	HCSF1 was centrally coordinated; HCSF2 used local site coordinators; Turtle Island Tales increased integrated recruitment and delivery into existing partner roles (health educators, teachers, school staff)	Local implementer meeting minutes, notes, emails	Reduce parallel research infrastructure, support community-led implementation	Community partners with research team support	Governance	Support community fit and local ownership	Maintenance, feasibility
Research incentives and retention strategies reduced or localized	*Turtle Island Tales*/*planned*	Family payments and text-message reminders used in earlier research trials were discontinued; local raffles or small gifts were used where communities chose to support evaluation completion	Local implementer meeting minutes, notes, emails	Reduce cost/burden, align procedures with community-led implementation priorities	Community partners with research team support	Structure, Governance	Support community fit and local ownership	Maintenance
Digital engagement shifted from Facebook groups to Instagram	*HCSF2* to *Turtle Island Tales*/*planned*	Invitation-only site-specific Facebook groups were replaced by public Facebook and Instagram accounts	External context/trends	Reflect shifts in social media use and broaden reach beyond enrolled participants	Research team, communications partners	Governance	Promote family-level wellness behaviors	Reach, dissemination
Training and manuals revised for broader delivery	Across all versions/*planned*	Training evolved from one-on-one training of dedicated site coordinators to group training for community/school staff; implementation manuals refined and expanded	Study and training protocols, community feedback via meeting minutes and in-person conversations during trainings	Prepare implementers for broader community delivery; support consistency across sites	Research team guided by community feedback	Structure	Support community fit and local ownership	Adoption, maintenance, fidelity

a All adaptations were either planned between program iterations or incorporated as part of prospective implementation design. No reactive, mid-iteration implementation adaptations were identified from available records.

b When decision processes or evidence sources could not be fully reconstructed from available records, the table reports the available source or process without inferring missing details.

c Core functions correspond to Table 2.

d Implementation outcomes are listed as intended outcomes based on available program records and team consensus; they were not evaluated as causal effects in this retrospective analysis.

### Governance adaptations

Governance evolved from more centralized advisory oversight during efficacy trials to formal cultural review and increased local decision authority during dissemination. During *HCSF1* and *HCSF2*, Community Advisory Boards (CABs) supported study conduct, recruitment, and progression and preserved the core function of community fit and local ownership. *HCSF1* CAB composition, meeting frequency, and broader community impact have been previously reported [23]. One *HCSF1* community also participated in *HCSF2*, which saw continued expansion of their CAB beyond study involvement to wider community-engaged work [24]. Similar documentation is not available for the other *HCSF2* community-specific CABs; therefore, meeting frequency and the full sequence of CAB decisions were not reconstructed here. In general, because implementation increased from four communities in one state in *HCSF1* to one community in each of five states in *HCSF2*, governance involved monthly virtual meetings with the five local community program coordinators. This approach was noteworthy given that virtual meetings were not part of routine community-engaged work at this time (2012–17). During the transition to *Turtle Island Tales*, community governance expanded through the creation of a cross-community Native Advisory Group that included five members with expertise in social work, community health, cultural education, family healthcare, sustainable development, Cooperative Extension, and the cultural practices of their respective communities. The group met virtually approximately 26 times from May 2017 to June 2018 and *ad hoc* through October 2025 to review draft lesson materials, visual design elements, lesson names, multimedia development, cultural framing, and to propose new lesson topics. Additional feedback was elicited through email. Recommended adaptations were included when they were consistent with core functions and community priorities. Integration of the recommendations was completed by the research team with iterative review by group members. Decisions were made by group consensus. This process was intended to strengthen cultural appropriateness and prepare program materials for broader dissemination while preserving the core function of grounding the program in Native culture, strengths, and intergenerational knowledge sharing.

As intervention delivery became more integrated with existing community systems during *Turtle Island Tales* implementation (described below for *strategy adaptations*), governance adaptations included expanded local implementer discretion. Local implementers include Head Start and school personnel (teachers, curriculum directors, administrators, staff), Cooperative Extension agents, and other community health personnel. Beginning in 2023, monthly community-specific meetings have been held, and adaptations are tracked through a checklist at each meeting. A cross-community Project Steering Committee meets quarterly to provide additional feedback. This shift allowed optional components to be adapted to local context while supporting feasibility, adoption, and sustainability. For example, although newsletters were centrally produced in *HCSF1* and *HCSF2*, their use in *Turtle Island Tales* has been determined locally. Some communities have integrated *Turtle Island Tales* updates into existing newsletters, while others have not included newsletters at all, reflecting local control of optional structural components. Although these governance adaptations influenced implementation strategies such as recruitment, delivery, newsletters, and evaluation incentives, they were coded primarily as governance because the central change concerned who had authority to review, approve, and localize adaptations.

### Content adaptations

Content adaptations expanded behavioral topics and strengthened cultural framing while preserving core functions related to family health promotion. Between *HCSF1* and *HCSF2*, two new lessons were added: “Sleep Tight,” focused on child sleep hygiene, and “Maintaining Harmony,” addressing adult stress management. These changes were directly informed by trial findings: parent-completed 24-hour dietary recalls during *HCSF1* revealed late-night eating among young children in the study, while participant focus groups highlighted stress as a barrier to family wellness. Given that sleep and stress are critical obesity risk factors, content was developed to address these topics within the intervention.

Between *HCSF2* and *Turtle Island Tales*, adaptations expanded emotional regulation content and strengthened cultural framing. “Feeling Your Feelings” was developed as a child-focused emotional regulation lesson, and “Family Traditions” was developed to more intentionally highlight family and community traditional practices. To accommodate these additions, overlapping nutrition lessons on fruit and added sugar intake were consolidated into a single “Naturally Sweet” lesson, and a fast-food–focused lesson (“Fast Lane to Health”) was removed, reflecting community preference for messaging that promotes nutrient-dense foods rather than restricting less healthy options.

Additional cultural framing elements were integrated during the transition to *Turtle Island Tales*. The Native Advisory Group prompted the inclusion of the medicine wheel as a core organizing concept in each *Turtle Island Tales* lesson, highlighting the interconnectedness of physical, spiritual, emotional, and mental health. Lesson materials depict which components are the focus for each lesson (e.g. the “Outdoor Adventures” lesson highlights physical and spiritual health). Grandmother quotes were added to each lesson to reinforce themes and values, and the Seven Grandfather teachings were incorporated throughout program materials, including coloring sheets and short films. The Native Advisory Group also recommended revisions to lesson names to better reflect lesson intent and cultural framing (e.g. “Fun Family Fitness” became “Outdoor Adventures”).

### Structure adaptations

Structure adaptations changed how intervention content was packaged and delivered. A substantial structural adaptation has been the change in delivery methods across iterations. *HCSF1* tested intervention delivery via home visits by community mentors versus mailed delivery of intervention materials. Home visits were initially prioritized for their personal connection, but they proved costly, difficult to schedule, and were not associated with improved outcomes compared to mailed delivery. As a result, *HCSF2* adopted mailed delivery as the standard modality. *Turtle Island Tales* further diversified delivery methods through an intentional focus on community-selected delivery structures, with most communities choosing to distribute materials through Head Start (a federally funded early childhood education program) and school classrooms by sending them home in children’s backpacks or cubbies. Some communities have experimented with hybrid approaches, such as delivering program materials to families at home during a separate home-visiting program. Other communities have distributed first kits during back-to-school or parent-night events or have sent home a packet of several kits over winter or summer break. In *HCSF1* and *HCSF2*, families could be enrolled year-round, with materials sent on a rolling basis. For *Turtle Island Tales*, we aligned recruitment with the school year to support classroom-based delivery, beginning each August or September, which streamlined implementation and supported community planning.

Another important structural adaptation was changing the program’s name to *Turtle Island Tales* to acknowledge ‘Turtle Island’ as the term for North America used by many Native American communities and to emphasize the importance of storytelling as a culturally relevant method of sharing knowledge. The Native Advisory Group also guided engagement with a graphic design firm, Art & Sons (Madison, WI), to create a logo and design that were consistent with the target of the intervention to reach Native American communities broadly. These structural adaptations altered how content was packaged and delivered, without modifying the educational objectives themselves. The logo, graphics, and color scheme were applied to the original intervention materials to update the printed parent lessons and support items (e.g. Fig. S1). Additionally, the group helped with the creation of new characters for the program: “Grandma” and “Igmu,” who is Grandma’s bobcat puppet “grandson.” These characters are featured throughout the program materials and in short films developed for *Turtle Island Tales*, which are available on the program website [25]. The films depict Grandma communicating the seven Grandfather teachings of love, respect, honesty, truth, wisdom, bravery, and humility to Igmu, as well as spiritual and cultural traditions of various regions of Turtle Island. The use of a Native grandmother to model healthy behaviors with her grandson aligns with Native values of elders passing on knowledge for future generations and supports the related core intervention function. The characters are used to maintain consistent branding across the materials, website, social media, and videos. For example, a cartoon version of Igmu is included throughout all lessons and some support items (e.g. stickers, coloring sheets).

### Strategy adaptations

Strategy adaptations focused on embedding implementation within existing community systems. Implementer training has remained a cornerstone strategy throughout all versions, with instructor manuals refined and expanded in each iteration. Recruitment strategies have shifted substantially. *HCSF1* and *HCSF2* relied heavily on community-wide recruitment strategies, such as in-person recruitment at community events and through flyers and social media. For *Turtle Island Tales*, these strategies were intentionally replaced with classroom-based recruitment through Head Start and schools in which one or more classrooms were selected for all children to receive the program using an opt-out enrollment approach. The shift in recruitment parallels the growth of community partnerships over time: *HCSF1* was centrally coordinated, *HCSF2* employed local site coordinators for recruitment and enrollment, and *Turtle Island Tales* integrated recruitment into existing roles of community partners (e.g. nutrition educators) to embed the program within established systems rather than creating parallel structures. The classroom-based recruitment approach was intended to promote broader reach and reduce burden on both families and program staff by eliminating the need for intensive individual-level recruitment. This strategy was also employed to address rising postage costs for mailing program materials and challenges in tracking participant addresses.

Financial incentive and digital engagement strategies also shifted. In *HCSF1* and *HCSF2*, families were compensated every 6 months for surveys to encourage retention as part of the research process. As the program moved toward community-led implementation, financial incentives were discontinued, which was intended to reduce costs and support program sustainability. For *Turtle Island Tales*, local implementers have created strategies to incentivize families to complete program evaluations, such as entry into gift card drawings, reflecting the shift toward sustainable community practice. For digital engagement, *HCSF2* relied on Facebook groups managed by a central study coordinator and local site staff to share recipes, wellness tips, and reminders. By *Turtle Island Tales*, this strategy transitioned almost entirely to Instagram, reflecting demographic shifts in social media use and aligning with the program’s broader dissemination goals. Importantly, Instagram content was made public, with the intent of broadening program reach beyond current study participants.

## Discussion

This article used *HCSF/Turtle Island Tales* as a longitudinal case example to document how a community-engaged intervention was adapted across governance, content, structure, and strategy during the transition from efficacy testing toward broader implementation. We described the rationale for adaptations across three successive program iterations, clarifying why changes were made and how they were intended to preserve core functions using a community-engaged, principle-guided adaptation [17, 22].

Governance adaptations were useful for distinguishing community-engaged decision-making processes from the downstream content, structure, and strategy adaptations that those processes produced [19, 26]. We conceptualized CABs and the Native Advisory Group not as discrete implementation strategies but as community-engaged governance processes [26] through which implementation strategies and intervention adaptations were selected, adapted, prioritized, monitored, and revised over time. From this perspective, the relevant dissemination and implementation functions of the CABs or Native Advisory Groups are captured by what each group was authorized to do [19]. CABs and advisory groups may support specific dissemination and implementation strategies (e.g. recruitment, training, delivery, evaluation procedures), but their primary contribution in long-term CBPR may be shaping who has authority to make adaptation decisions and how those decisions are reviewed and incorporated across the lifetime of the partnership.

Content adaptations expanded the range of topics, language, stories, and cultural framing while preserving the core family wellness function, consistent with cultural tailoring, the function/form distinction, and with modifications described in FRAME and the Adaptome [13, 14, 16, 17, 22]. Qualitative findings from prior trials supported the acceptability of the intervention’s family-centered content. These findings also suggested possible mechanisms, such as increased family time, shared reading, and co-engagement around stories, through which content adaptations may support behavior change. These findings informed decisions to preserve core functions while adapting intervention forms [27].

Structural adaptations were most useful for showing how the program’s delivery forms could change while preserving core functions. The program shifted from home visits (*HCSF1*) to mailed distribution (*HCSF2*) and then to classroom-based distribution with local variations (*Turtle Island Tales*). Further, distribution timing to align with the school-year calendar rather than rolling enrollment was intended to streamline implementation and support planning. Changes to branding using culturally resonant materials with consistent characters and graphics were intended to increase exposure to and comprehension of the intervention, as well as support implementation outcomes such as reach and fidelity [28, 29]. These also map to FRAME categories for delivery mode, personnel, and timing/dose, as well as scale-up/scale-out guidance [17].

Strategy adaptations were identified as any action to improve dissemination and implementation outcomes (i.e. reach, adoption, fidelity, and maintenance) that were often a result of shifting structure. A good example of adaptations to a strategy intended to improve reach is found in the changes to recruitment activities that moved from intensive individual recruitment to a classroom-based opt-out approach, a shift that may be relevant to other settings to increase reach and reduce burden and workload [30]. Adaptations that leverage existing staff roles and routines instead of project-specific positions may support adoption and maintenance while preserving core intervention functions [31]. Changes to strategies around financial incentives, training cadence and manuals, and social media align with dissemination and implementation targets of reach, adoption, implementation quality, and maintenance and with CEDI guidance on identifying ready partners and systems for dissemination [27].

One of the challenges we encountered during this process was distinguishing between governance, content, structure, and strategy adaptations. Even among the coauthors, there were spirited debates on categorizing adaptations, such as designing for dissemination (i.e. is that a strategy or changes to structural characteristics?) [32]. For example, the development of culturally resonant characters and visuals was primarily categorized as a structural adaptation because it altered how content was packaged and delivered; however, these characters were also designed to create excitement and buy-in from families and children, and from an alternative perspective, could reasonably be considered a dissemination or engagement strategy intended to support reach and uptake. In our efforts to clarify the distinction among adaptation categories developed in the context of CBPR, we concluded that some adaptations may reasonably be assigned to multiple categories. Consistent with others in the field, we considered strategies as actions [33], but we also defined governance in the context of CBPR codesign processes as related to, often driving, but also distinct from content, structure, and strategy [34, 35].

Systematically documenting adaptations that occur as interventions transition from efficacy testing to effectiveness, dissemination, and scale-up may advance the science of implementation and intervention adaptation. To support these advances, we suggest a few recommendations for others using CBPR, CEDI, or other participatory approaches. First, we recommend using the categories of adaptation to include governance, content, structure, and strategy. Governance may be especially useful when decision authority, community review, or local discretion change over time. Second, when reporting governance, investigators should describe not only whether a CAB or advisory group was used but also its composition, decision scope, authority, input process, adaptation role, timing, accountability processes, and evolution. Third, when coding adaptations, we encourage investigators to allow adaptations to “walk the line” with coding for primary and secondary categories of adaptation. This is likely to resolve debates while also contributing to an understanding of the complexity of adaptation decisions and relationships. Fourth, we recommend relying on available adaptation evidence, including meeting minutes, IRB protocol modifications, training guides, intervention iterations, and archived emails. We found that using these materials and, in particular, electronic versions of each intervention iteration, allowed for a relatively accurate characterization of changes that occurred over almost two decades. At the same time, investigators should clearly identify which adaptation elements cannot be reconstructed retrospectively rather than inferring undocumented details.

### Limitations

As a retrospective review, some important specifications of the adaptations proposed by models like FRAME and FRAME-IS are difficult to capture (e.g. precise timing, decision-maker, intent) and may introduce inaccuracies in reporting. The tools we used to conduct this review varied across the project and over time, and some details, such as the full sequence of decision-making for each adaptation, could not be reconstructed. We also did not evaluate the causal effects of individual adaptations on effectiveness or implementation outcomes. Although it is a strength to have a codesigned health promotion intervention with Native American communities, our evaluation represents a single intervention, and generalizability to other populations, interventions, and settings should be tested.

### Implications for implementation science

This study illustrates one approach to systematically documenting adaptations across multiple stages of intervention development, testing, and dissemination and implementation. Attention to the cumulative and layered nature of adaptations is particularly critical for community-engaged and equity-oriented interventions designed to evolve over time with changing community context. Organizing adaptations into governance, content, structure, and strategy provides one pragmatic approach for distinguishing modifications to intervention forms from those intended to improve implementation outcomes while preserving core functions. Our findings also suggest that documenting how the governance of adaptation decisions may be as important as documenting the adaptations themselves. For community-engaged interventions, this includes documenting not only whether advisory groups were used but also what governance functions they performed and how decision authority changed over time. This also extends to the potential for sustained implementation, which may depend less on preserving fixed intervention packages than on preserving the collaborative processes that allow interventions to evolve while maintaining core functions. Beyond the specific evolution of *HCSF/Turtle Island Tales*, this case example demonstrates how retrospective documentation of adaptations can complement existing adaptation frameworks by capturing the cumulative changes that occur as interventions mature through successive stages of implementation.

### Conclusions

Organizing adaptations into governance, content, structure, and strategy was pragmatic and actionable for CBPR intervention scale-up. This categorization and reporting of adaptations have the potential to advance our understanding of what intervention components to hold constant or allow for variability during scale-up of health promotion interventions. The adaptation principles described here may inform similar community-engaged interventions, although their transferability to other settings should be evaluated prospectively. Systematic reporting of adaptations can contribute to the growing field of CEDI science and provide a replicable model for balancing preservation of core functions with adaptations to forms and strategies. This includes making clear not only what changed but also how community-engaged governance processes shaped adaptation decisions over time.

## Supplementary Material

ibag056_Supplementary_Data

## Data Availability

No human subjects data are reported in this article. Any data from the present work will be made available by emailing the corresponding author.

## Welfare of animals

This article does not contain any studies with animals performed by any of the authors.
